# Supplementary material for: Exploring the Potential of Lapatinib, Fulvestrant, and Paclitaxel Conjugated with Glycidylated PAMAM G4 Dendrimers for Cancer and Parasite Treatment
Source: Molecules. 2023 Aug 30;28(17):6334. doi: 10.3390/molecules28176334 (PMC10489794; doi:10.3390/molecules28176334)
Supplement: Supplementary file 1 [file molecules-28-06334-s001.zip › molecules-2535313-supplementary.pdf]

SUPPLEMENTARY MATERIALS

A

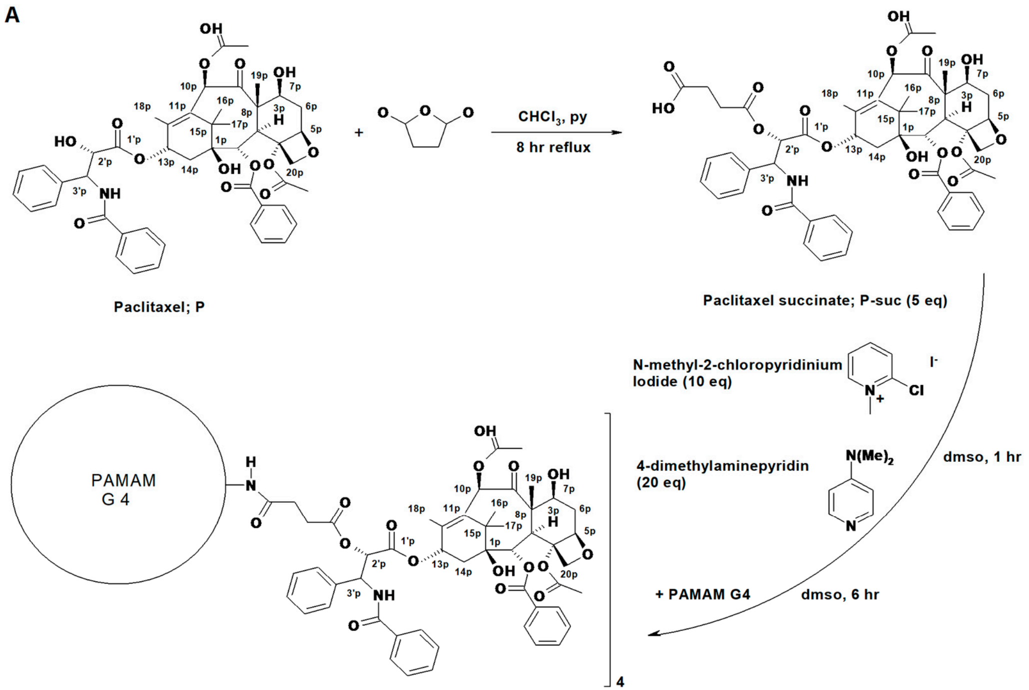

Figure S1 A: Scheme of synthesis of PAMAM G4 – Paclitaxel conjugate

B

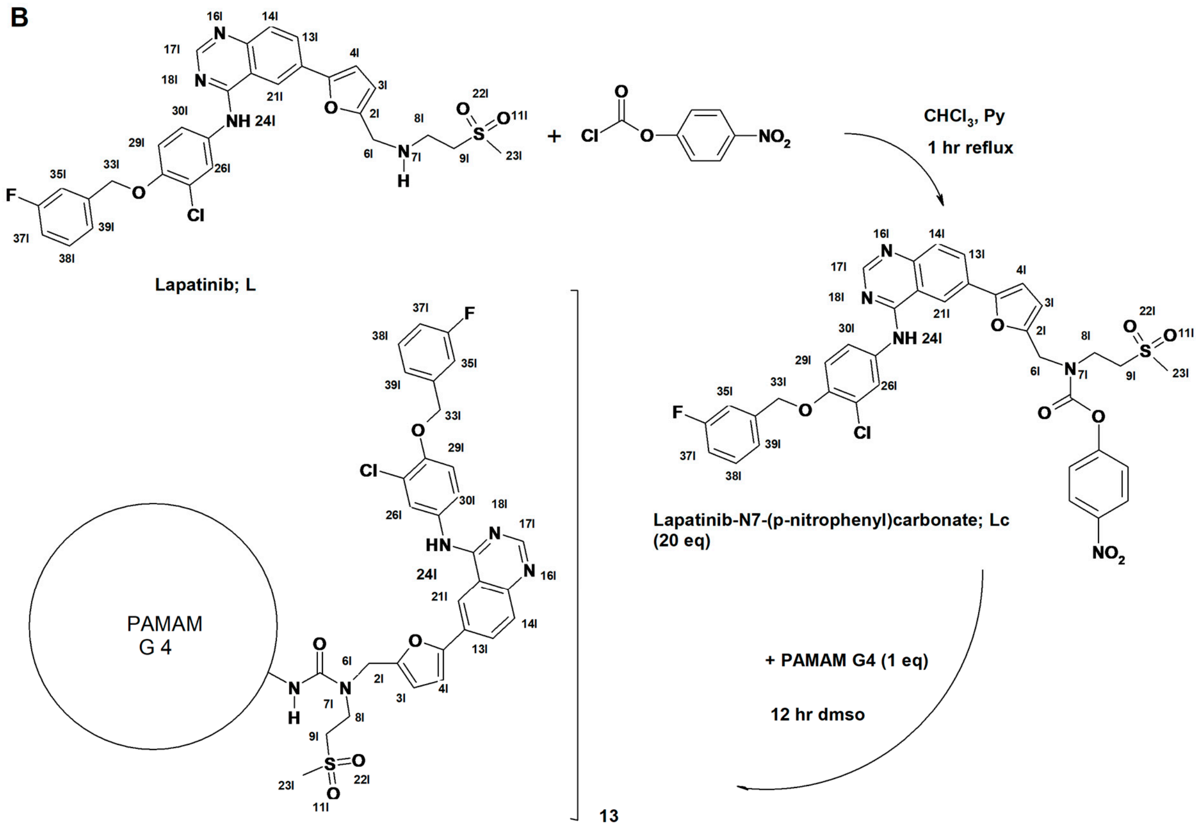

Figure S1 B: Scheme of synthesis of PAMAM G4 – Lapatinib conjugate

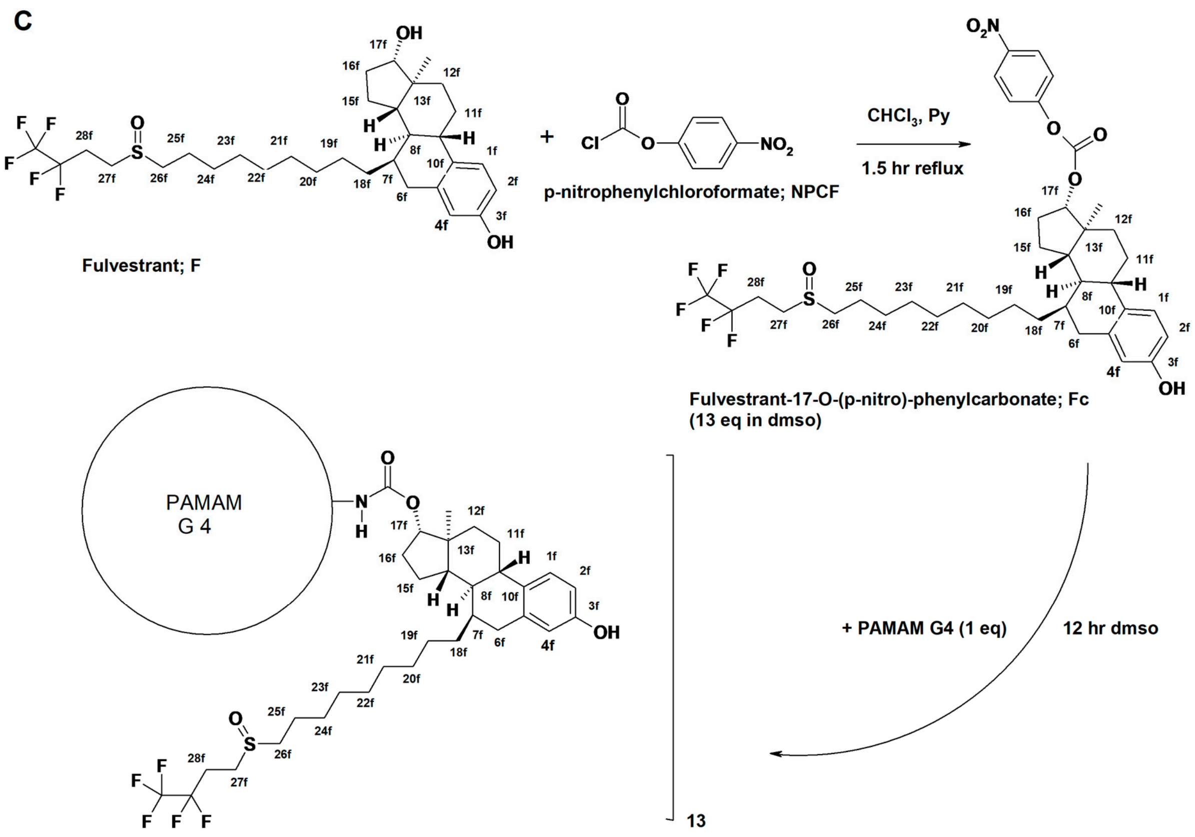

Figure S1 C: Scheme of synthesis of PAMAM G4 – Fulvestrant conjugate

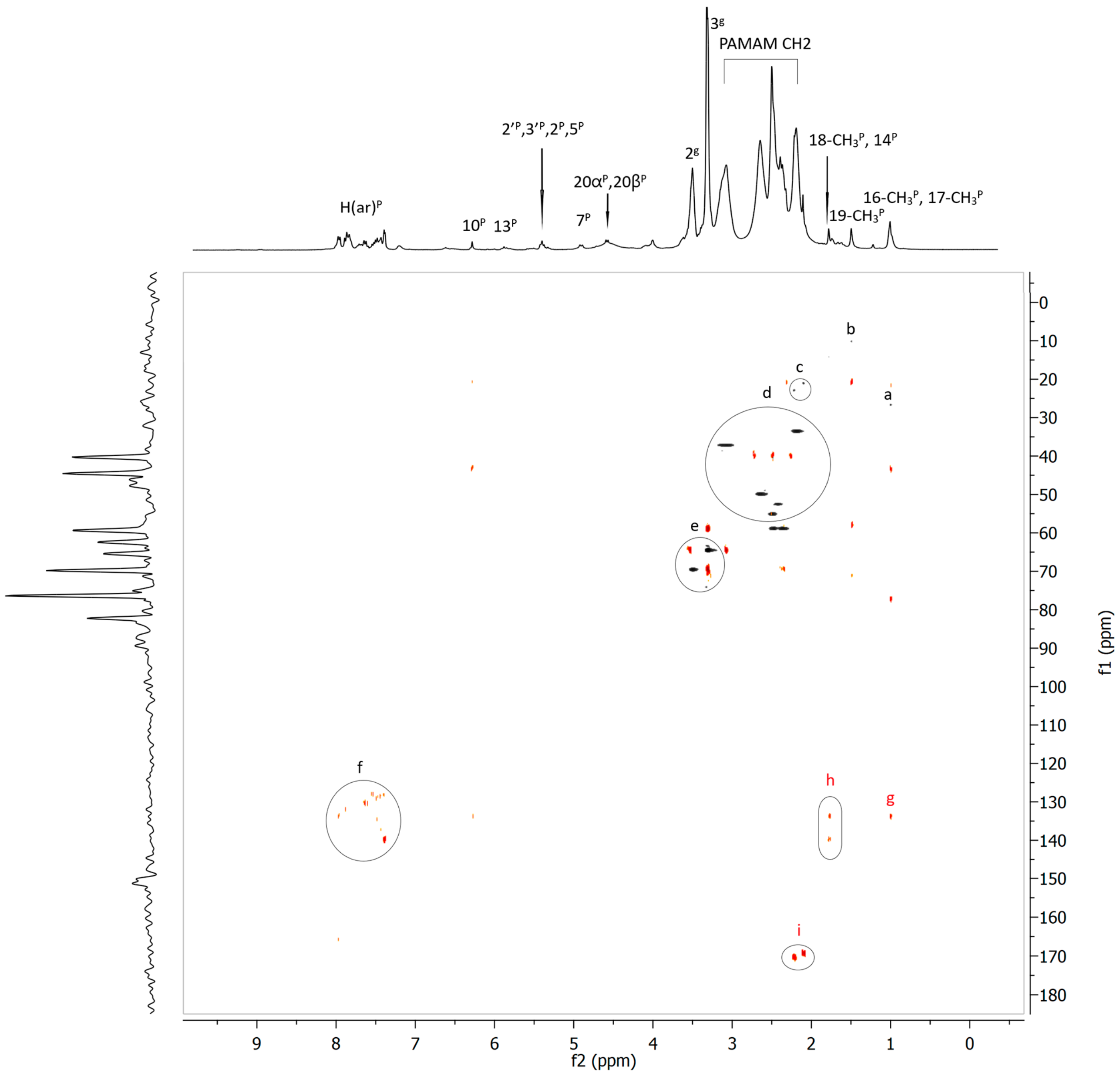

Figure S2 A: Combined HSQC/HMBC map of PAMAM G4*[4P109gl] in DMSO-d_6

The relevant one bond coupling peaks are shown in grey scale
as follows:

a: 17-CH_3^P/17C^P or 16-CH_3^P/16C^P ; b: 19-CH_3^P/19C^P ; c: 10H^P-OAcH / 10^P-OAcC and 4H^P-OAcH / 4C^P-OAcC

The significant HMBC coupling peaks are represented in red/yellow scale as follows:

g: 16-CH_3^P/ 11C^P or 17-CH_3^P / 11C^P ; h: 18-CH_3^P / 11C^P and 12C^P ; i: 10H^P (OAcH) / C(O)^P and 4H^P (OAcH) / C(O)^P

d represents HMBC and HSQC cross peaks of PAMAM G4 protons and carbons.

As „e" HMBC and HSQC cross peaks 2H^g/2C^g ; 2H^g/3C^g ; 3H^g/3C^g ; 3H^g/ 2C^g are presented.

A group of HMBC/HSQC aromatic cross-peaks of P residues is assigned as „f".

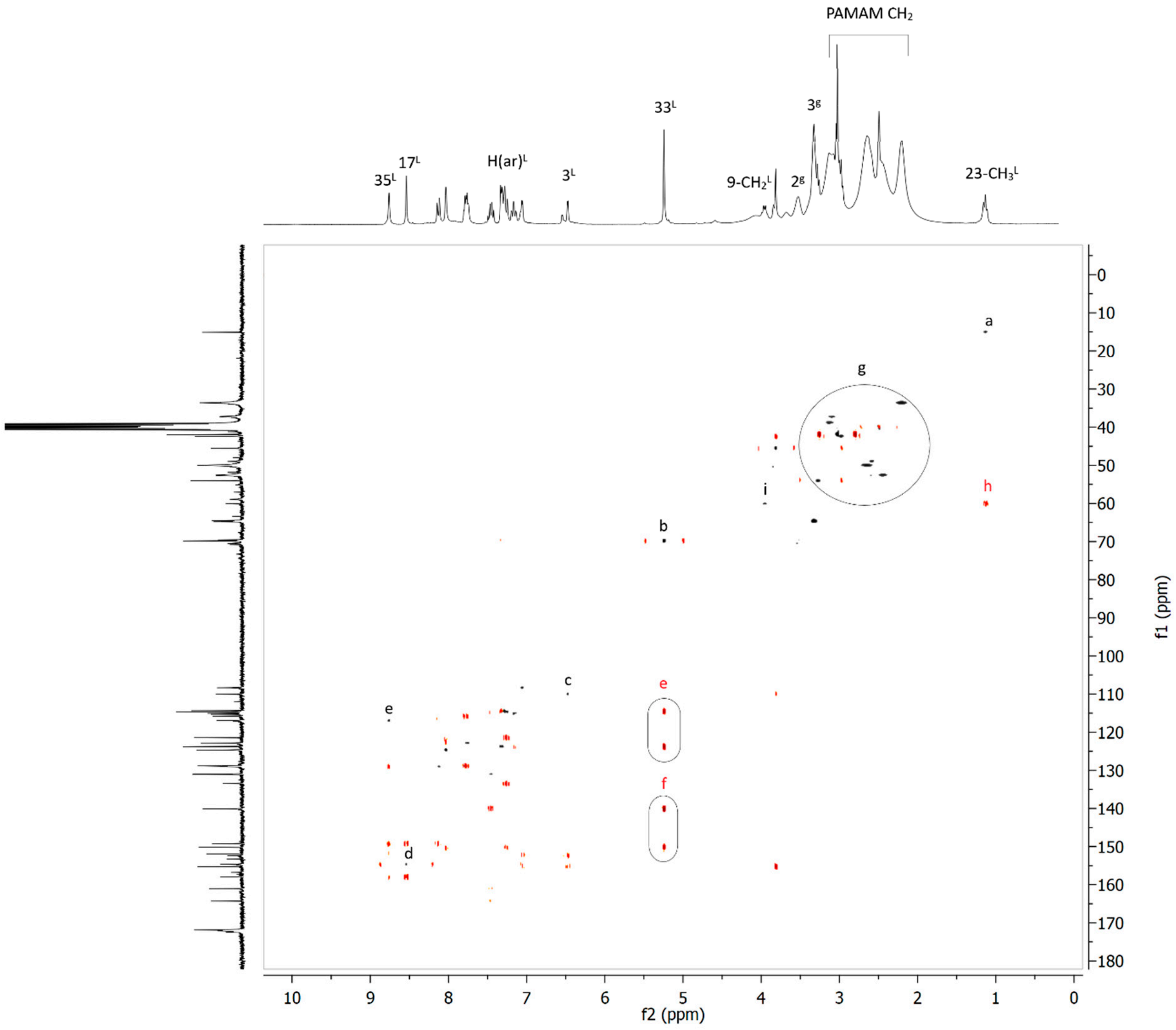

Figure S2B: Combined HMBC/HSQC map of $G4^{*13L85gl}$ in DMSO-d_6

The relevant HSQC cross peaks are shown in grey-scale as follows: a: $23\text{-}CH_3^L/23C^L$; i: $9\text{-}CH_2^L/9C^L$; b: $33H^L/33C^L$; c: $3H^L/3H^C$; d: $17H^L/17C^L$; e: $35H^L/35C^L$

The significant HMBC coupling peaks are represented in red/yellow scale as follows:

h: $23\text{-}CH_3^L/9C^L$; e: $33H^L/35C^L$ and $33H^L/39C^L$; f: $33H^L/28C^L$ and $33H^L/40C^L$

g represents group of HMBC and HSQC cross-peaks of PAMAM G4 protons and carbons.

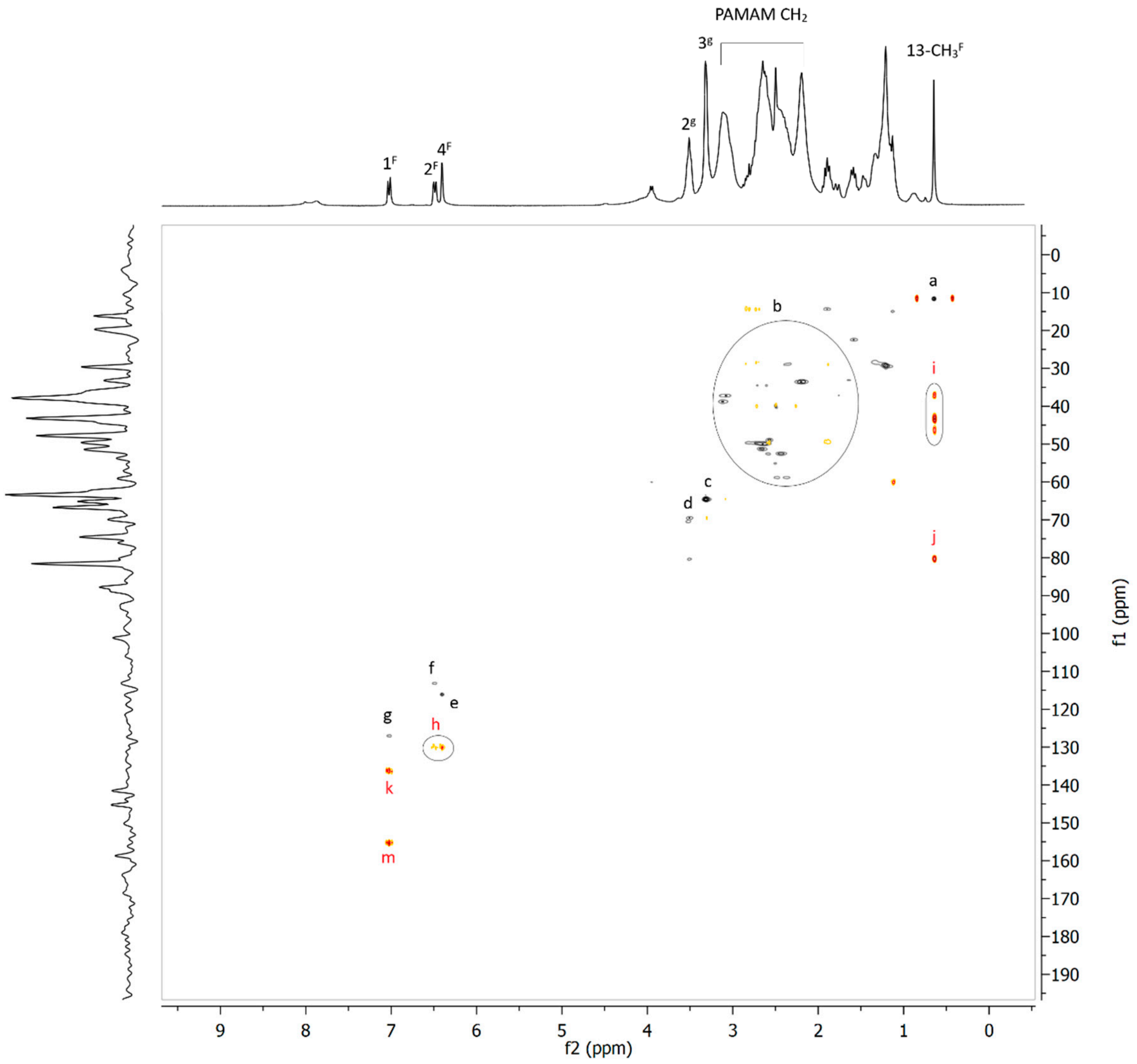

Figure S2C: Combined HMBC/HSQC map of $G4^{*13F64gl}$ in DMSO-d_6

The significant one bond coupling peaks are shown in grey-scale as follows:

a: 13-CH_3^F/$13C^F$; c: $3H^g$/$3C^g$; d: $2H^g$/$2c^g$; e: $4H^F$/$4C^F$; f: $2H^F$/$2C^F$; g: $1H^F$/$1C^F$

The crucial HMBC coupling peaks are represented in red/yellow scale as follows:

i: 13-CH_3^F/$14C^F$,$12C^F$,$11C^F$; j: 13-CH_3^F/$17H^F$; h: $4H^F$/$5C^F$ and $2H^F$/$5C^F$; k: $1H^F$/$10C^F$; m: $1H^F$/$3C^F$

As „b” a group of HMBC and HSQC cross peaks of PAMAM G4 protons and carbons is represented.

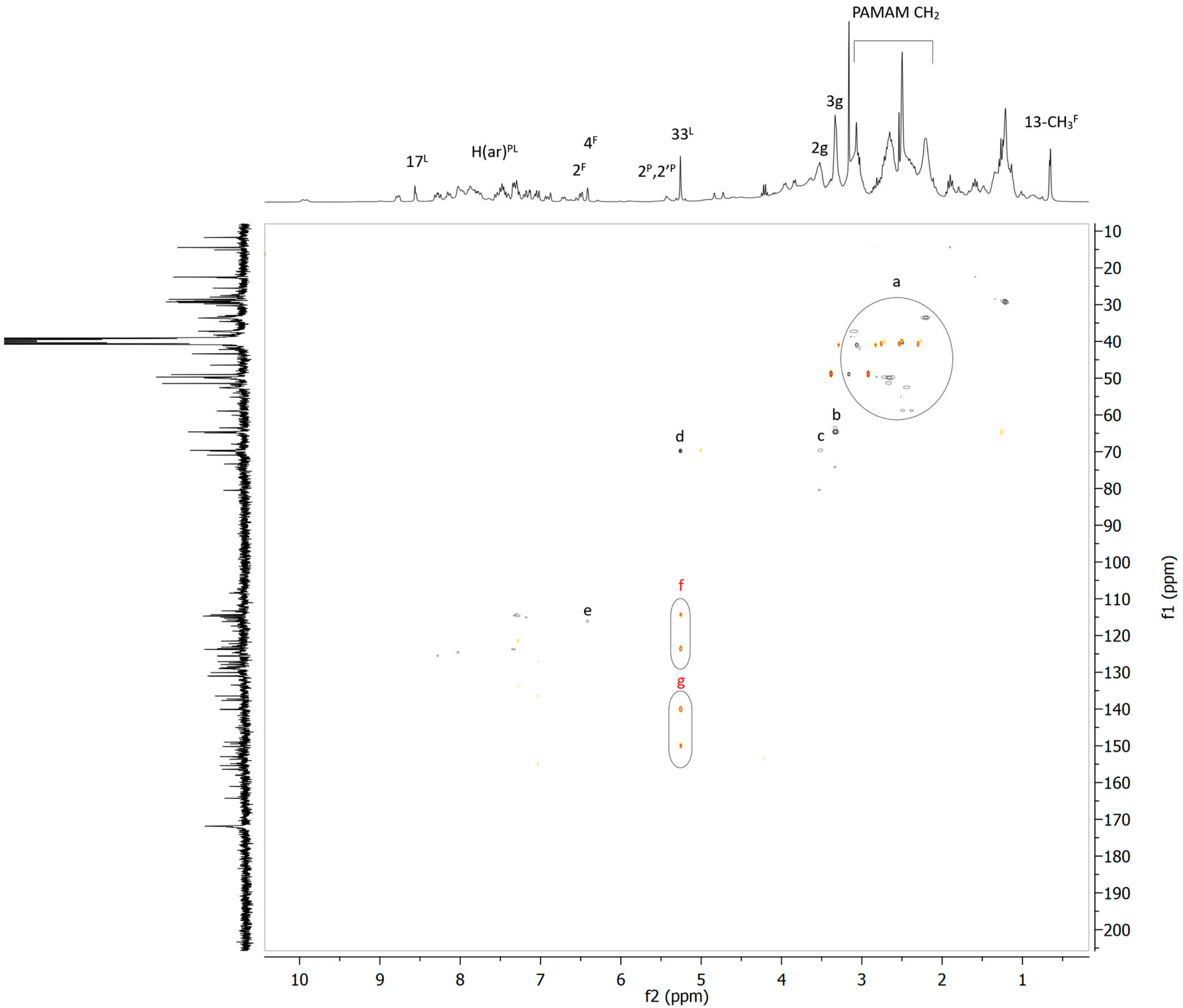

Figure S2D: Combined HMBC/HSQC map of PAMAM G4*^{4P11F11P74gl} in DMSO-$d_{6.}$

The crucial one bond coupling cross peaks are represented in grey-scale as follows:

b: $3H^g/3C^g$; c: $2H^g/2C^g$; d: $33H^L/33C^L$; e: $4H^F/4C^F$

The relevant HMCQ coupling peaks are shown in red/yellow scale as follows:

f: $33H^L/35C^L$ and $33H^L/39C^L$; g: $33H^L/28C^L$ and $33H^L/40C^L$

As „a" a group of HMBC and HSQC cross peaks of PAMAM G4 protons and carbons is represented.

Solution		aqueous				0.05 M acetate buffer pH 5		
**Species ↓**	d(V) (SD) [nm]	d(N) (SD) [nm]	PDI (SD)	ζ (SD) [mV]	d(V) (SD) [nm]	d(N) (SD) [nm]	PDI	ζ (SD) [mV]
**G4*[P]**	154.73 (4.29)	99.20 (7.28)	0.134 (0.016)	13.57 (0.43)	156.26 (2.77)	94.62 (4.32)	0.166 (0.014)	33.26 (1.21)
**G4*[L]**	162.06 (1.80)	113.13 (4.64)	0.112 (0.014)	40.29 (0.74)	a)	58.19 (15.84)	0.238 (0.009)	47.70 (3.29)
**G4*[F]**	233.83 (9.02)	138.23 (6.24)	0.155 (0.022)	38.02 (0.54)	b)	155.37 (8.76)	0.508 (0.044)	45.23 (1.73)
**G4*[PL]**	147.89 (1.86)	95.20 (4.46)	0.125 (0.010)	19.92 (0.53)	132.02 (1.59)	89.22 (6.33)	0.126 (0.019)	32.67 (1.57)
**G4*[PF]**	174.82 (2.10)	113.41 (5.48)	0.128 (0.015)	29.29 (0.72)	125.26 (1.72)	81.60 (1.67)	0.134 (0.010)	33.83 (1.04)
**G4*[PFL]**	174.38 (3.71)	105.47 (5.39)	0.159 (0.018)	34.33 (0.65)	165.00 (1.83)	118.78 (4.66)	0.093 (0.020)	35.32 (0.87)

Table S1. The values of ζ potential and number- and volume-averaged diameter of conjugate molecules (*d*(N) and *d*(V), respectively), in water and in 0.05 M acetate buffer pH 5 (measured within 1 h after dissolving). SD, standard deviation.

a) d(V) of **G4L** pH 5: 106.6 (46.8); 39.353 (31.1%); 207.9 (100 %); 362.5 (51.1%); 217.7 (67%); 56.71 (38.4%); 49.9 (26.9%); 196.8 (92.3 %); 82.26 (50.4 %); 46.30 (19.9%); 88.29 (47.8%); 82.67 (50.8%); 256.1 (61.6%); 221.6 (73.1%); 282.1 (49.6%); 95.45 (33.8%); 300.5 (46.2%); 305.3 (52.2%); 304.9 (49.2%)

b) d(V) of **G4F** pH 5: 182.7 (27.7%); 206.3 (29.3%); 183.5 (22.1%); 210.3 (18.7%); 186.5 (19.8%); 183.0 (29.0%); 179.8 (22.7%); 191.1 (28.2%); 165.3 (23.5%); 707.9 (72.3%); 818.2 (63.7%); 774.5 (69.0%); 1078.0 (76.1%); 954.3 (80.2%); 660.2 (66.7%); 770.4 (73.0%); 746.2 (71.8%); 715.2 (71.6%)

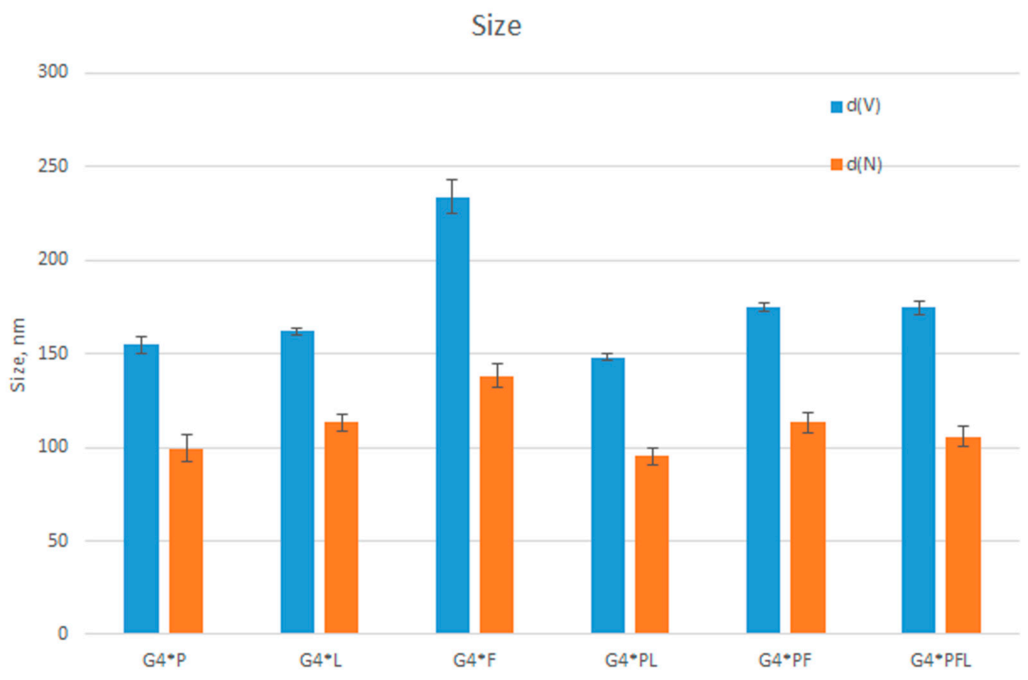

Figure S3: Hydrodynamic diameter averaged by volume (d(V)) and number (d(N)) of obtained conjugates in water

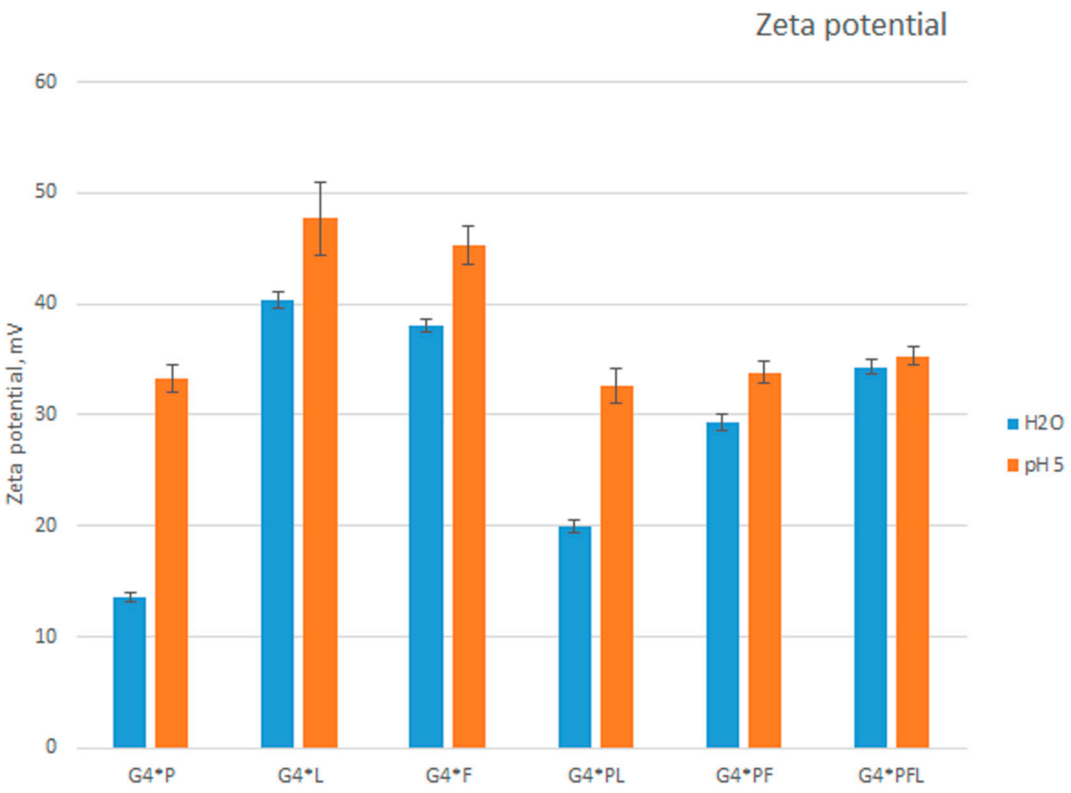

Figure S4: Zeta potential of obtained conjugates measured in water and in phosphate buffer pH 5

Figure S5: The raw data on size didstribution measurements of conjugates in water and in pH 5: A – G4P; B – G4PFL;
C – merged results for G4P, G4L, G4F, G4PFL and binary conjugates (not tested biologically); D: average d(V) of G4F in water and pH 5

A

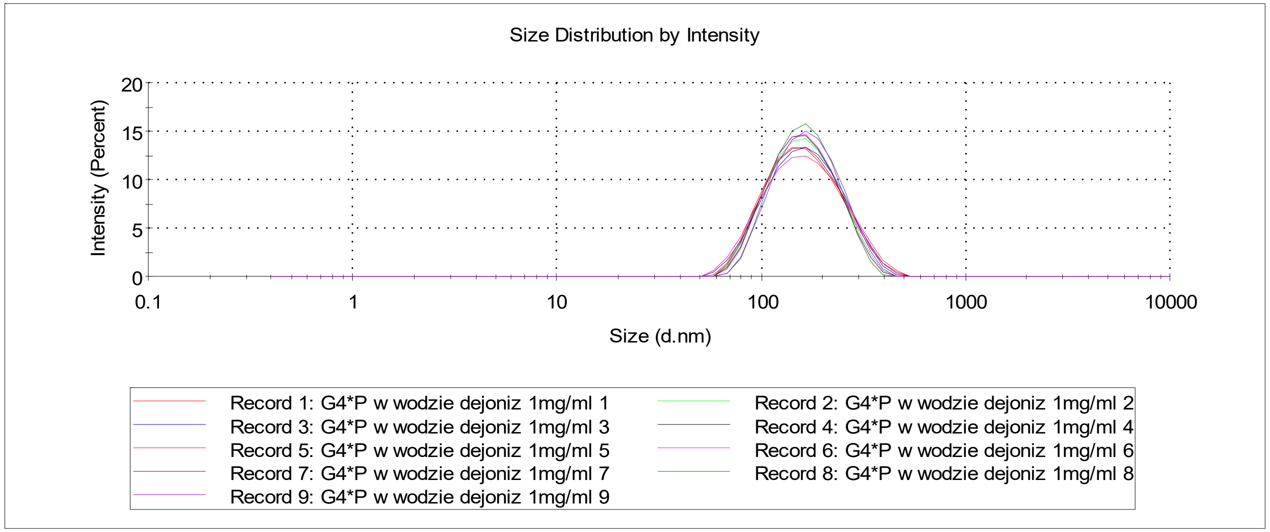

B

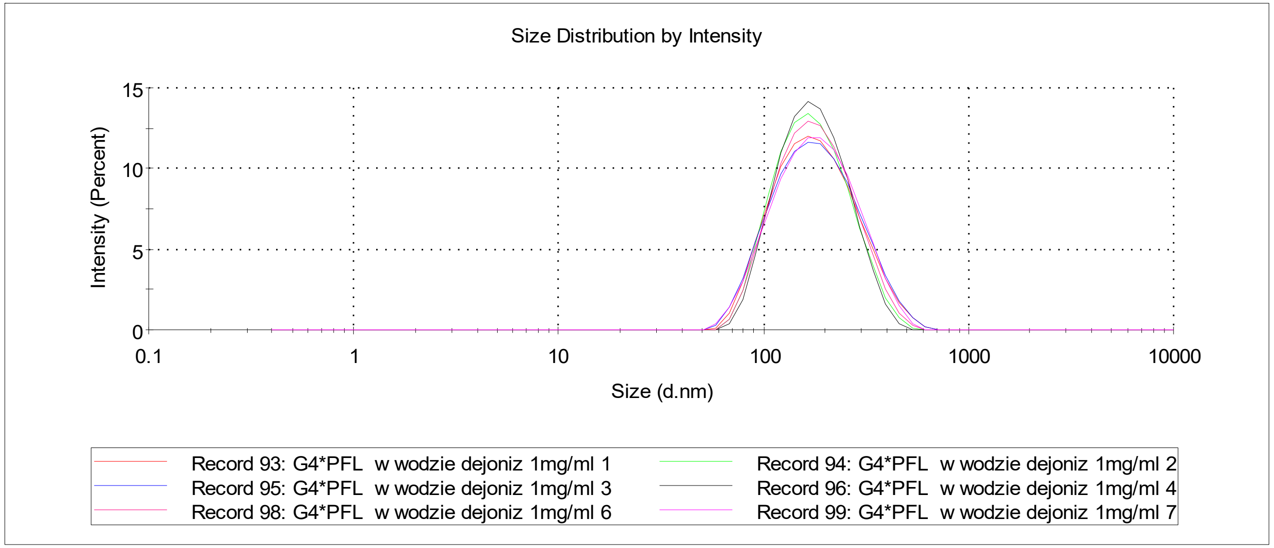

C

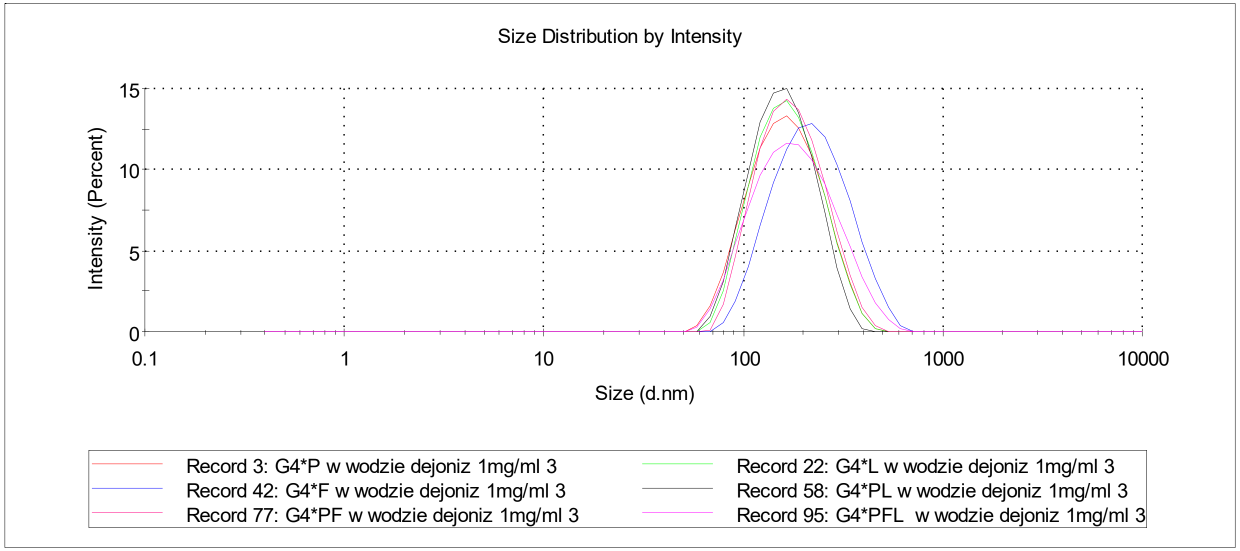
Size Distribution by Intensity
Intensity (Percent)
15
10
5
0
0.1
1
10
100
1000
10000
Size (d.nm)
Record 3: G4*P w wodzie dejoniz 1mg/ml 3
Record 42: G4*F w wodzie dejoniz 1mg/ml 3
Record 77: G4*PF w wodzie dejoniz 1mg/ml 3
Record 22: G4*L w wodzie dejoniz 1mg/ml 3
Record 58: G4*PL w wodzie dejoniz 1mg/ml 3
Record 95: G4*PFL w wodzie dejoniz 1mg/ml 3

D

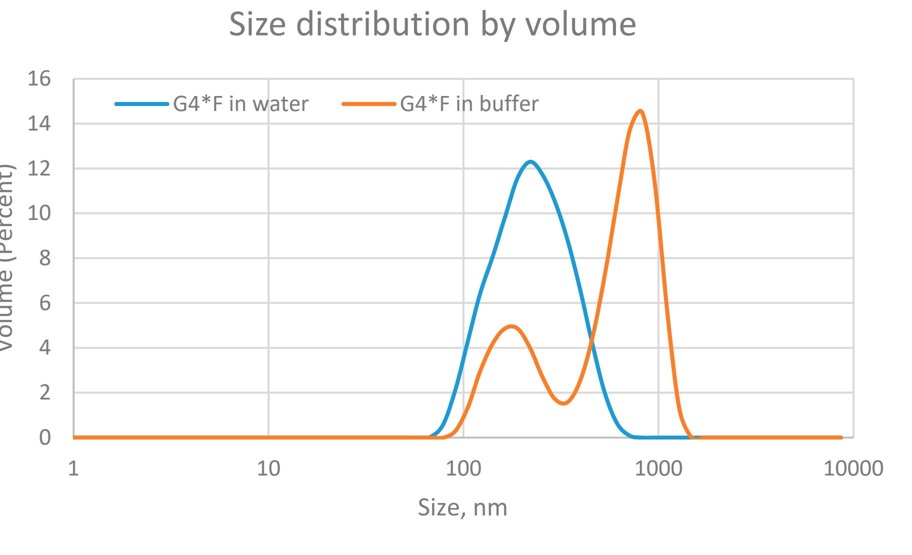
Size distribution by volume
Volume (Percent)
16
14
12
10
8
6
4
2
0
G4*F in water
G4*F in buffer
1
10
100
1000
10000
Size, nm
